# Immunotherapy for Hepatocellular Carcinoma: Current Advances and Future Expectations

**DOI:** 10.1155/2018/8740976

**Published:** 2018-03-26

**Authors:** Yingjun Xie, Yien Xiang, Jiyao Sheng, Dan Zhang, Xiaoxiao Yao, Yongsheng Yang, Xuewen Zhang

**Affiliations:** ^1^Department of Hepatobiliary and Pancreatic Surgery, Second Hospital of Jilin University, Changchun 130041, China; ^2^Jilin Engineering Laboratory for Translational Medicine of Hepatobiliary and Pancreatic Diseases, Changchun 130041, China

## Abstract

Primary liver cancer is a common kind of digestive cancers with high malignancy, causing 745,500 deaths each year. Hepatocellular carcinoma is the major pathological type of primary liver cancer. Traditional treatment methods for patients with hepatocellular carcinoma have shown poor efficacy in killing residual cancer cells for a long time. In recent years, tumor immunotherapy has emerged as a promising method owing to its safety and efficacy with respect to delaying the progression of advanced tumors and protecting postoperative patients against tumor relapse and metastasis. Immune tolerance and suppression in tumor microenvironments are the theoretical basis of immunotherapy. Adoptive cell therapy functions by stimulating and cultivating autologous lymphocytes ex vivo and then reinfusing them into the patient to kill cancer cells. Cancer vaccination is performed using antigenic substances to activate tumor-specific immune responses. Immune checkpoint inhibitors can reactivate tumor-specific T cells and develop an antitumor effect by suppressing checkpoint-mediated signaling. Oncolytic viruses may selectively replicate in tumor cells and cause lysis without harming normal tissues. Here, we briefly introduce the mechanism of immunosuppression in hepatocellular carcinoma and summarize the rationale of the four major immunotherapeutic approaches with their current advances.

## 1. Introduction

Primary liver cancer is the sixth most common type of cancer and the second most common cause of cancer-related deaths worldwide, with an extremely high malignancy such that the number of deaths (745,500) is similar to that of new cases (782,500) every year [1]. Hepatocellular carcinoma (HCC) is a predominant type of primary liver cancer. Traditional therapeutic approaches for HCC include radical or palliative liver resection, radioactive seed implantation, transarterial chemoembolization (TACE), radiofrequency ablation (RFA), and liver transplantation. Although these approaches effectively address local lesions, they fail to completely eliminate residual cancer cells, which lead to tumor recurrence and metastasis. In recent years, tumor immunotherapy has emerged as a promising method for inhibiting tumor progression, relapse, and metastasis [2]. The rationale of this method is to activate tumor-specific immune responses and disrupt immune tolerance by enhancing cellular or humoral immunity. To date, some immunotherapeutic drugs for treating hematological malignancies, melanomas, and lung cancers have been proven to be efficacious in phase III trials and have been approved by FDA. Furthermore, recently, studies on immunotherapeutic approaches for HCC are rapidly increasing. In this study, we briefly reviewed the mechanism underlying immunosuppression and summarized major immunotherapeutic approaches for HCC (Table 1).

## 2. Mechanism Underlying Immunosuppression in HCC

T cells are activated through a double signaling pathway that requires the interaction of T cell receptors (TCR) with major histocompatibility complex (MHC)/peptide complexes on antigen-presenting cells (APCs) and expression of costimulatory molecules (CMs) on T cells and APCs. Downregulation of MHC class I molecules on tumor cells induces impairment of tumor antigen processing and presentation [3, 4]. Furthermore, reduced expression of CMs, such as B7-1 and B7-2, in HCC [4] leads to T cell anergy.

Immune checkpoints normally protect humans from uncontrolled autologous immunity by preventing excessive activation of T cells. However, tumor cells can overexpress immune checkpoint molecules that bind their receptors on T cells and inhibit T cell activation. The upregulation of immune checkpoint pathways in most patients with HCC impairs the effector function of cellular immune responses [5–7].

Immunosuppression in HCC can also be achieved via impairment of CD4^+^ T cells [8]. MHC class II genes are among the most frequently expressed genes in HCC tumors, and overexpression of MHC class II molecules leads to CD4^+^ T cell anergy in the absence of suitable CMs [9]. Also, immunosuppressive cells, including T regulatory cells (Tregs) [10], myeloid-derived suppressor cells (MDSCs) [5], and regulatory dendritic cells (DCs) [11], are important immunosuppressive factors in cancer patients, and an increase in the number of immunosuppressive cells, such as Tregs, may contribute to disease progression and poor prognosis. A Th1/Th2-like cytokine shift in the liver microenvironment of HCC patients with venous metastases has been previously reported [12]. Moreover, the upregulation of anti-inflammatory/immunosuppressive Th2-like cytokines and downregulation of proinflammatory/immunogenic Th1-like cytokines in adjacent noncancerous hepatic tissues indicate that disordered immune responses in tumor microenvironments [13–15] are key predictors of HCC metastasis.

## 3. Adoptive Cell Therapy

Adoptive cell therapy (ACT) is an immunotherapeutic approach that kills cancer cells using patients' own lymphocytes. It functions by stimulating or loading autologous lymphocytes with cytokines or tumor antigens, cultivating them ex vivo and then reinfusing them into the patient [16–18]. Adoptive immunotherapy for HCC includes cytokine-induced killer (CIK) cells, tumor-infiltrating lymphocytes (TILs), natural killer (NK) cells, and chimeric antigen receptor (CAR) T cells. The feasibility and safety of ACT in patients with HCC have been evaluated in many experiments, thus laying a foundation for its clinical application.

### 3.1. CIK Cells

CIK cells are a heterogeneous MHC-independent cell population mainly comprising CD3^+^CD56^+^, CD3^+^CD56^−^, and CD3^−^CD56^+^cells [19–21]. CIK cells are derived from peripheral mononuclear cells and stimulated by IL-1, IL-12, interferon- (IFN-) *γ*, and anti-CD3 antibodies ex vivo [22]. In a phase III study of adjuvant CIK therapy after radical resection for HCC, patients were randomized to receive four cycles of CIK therapy or no treatment. The median time to recurrence (TTR) was 13.6 months in the CIK group and 7.8 months in the control group (*p* = 0.01), indicating the safety and efficacy with respect to prolonging TTR of CIK therapy in patients with HCC. However, there were no statistically significant differences between the groups in disease-free survival (DFS) and overall survival (OS) [23]. A combination therapy with CIK cells and valproate in mice demonstrated a synergistic effect in controlling tumor growth [24], warranting further assessment of this combination therapy through clinical trials. In addition, a meta-analysis of 693 patients with HCC demonstrated that a combination of dendritic cell- (DC-) CIK cells and TACE improves 1- and 2-year OS, overall response rate (ORR), disease control rate (DCR), and the quality of life [25].

### 3.2. TILs

TILs are derived from tumor tissues and are cultured and induced using IL-2 and anti-CD3 antibodies ex vivo [26–28]. Thus, reinfusion of autologous TILs, which possess tumor-specific immunity, may target multiple tumor antigens. Low toxicity of autologous TILs was verified in a phase I study involving patients with HCC, suggesting a novel treatment option [29]. However, this study included only 15 patients and lacked control groups, thus failing to prove the efficacy of TILs. To date, TILs have not been well characterized, mainly due to difficulties in purifying and expanding them.

### 3.3. NK Cells

NK cells belong to the innate immune system and can directly kill tumor cells and infected cells without preliminary sensitization or MHC restriction. However, they lack the ability to target tumor cells and can injure normal liver tissues. In a previous series of experiments, the cytotoxicity of NK cells against HCC cells was enhanced [30] by first generating a new hepatoma cell line, K562-mb15-41BBL, which achieved a more efficient stimulation of NK cells in vitro. Second, HCC cells exposed to 5 *μ*mol/L sorafenib for 48 h showed high sensitivity to NK cells. Finally, NKG2D, an engineered NK-cell-activating receptor, was tested in vitro and in mice. All of the outcomes were positive in increasing the cytotoxicity of NK cells, providing the possibility of further clinical trials for HCC.

### 3.4. CAR T Cells

**Figure 1:**
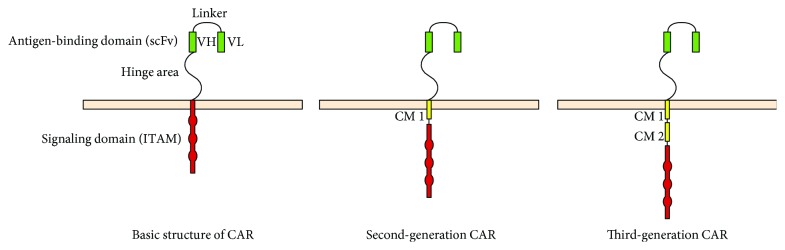
Structure of CAR.

CAR T cells are genetically modified T lymphocytes that specifically target tumor-associated antigens (TAAs) and kill cancer cells in a MHC-independent manner [31, 32]. CARs consist of three major components—the extracellular antigen-binding domain, the intracellular signaling domain, and the hinge area [33, 34] (Figure 1). The antigen-binding domain is a single chain fragment variable (scFv) region that comprises a heavy (VH) and a light (VL) chain derived from monoclonal antibodies (mAbs), which are connected by a linker fragment. The signaling domain involves immunoreceptor tyrosine-based activation motifs (ITAMs), such as CD3*ζ* and Fc*ε*RI*γ*. The above two domains are connected by the hinge area which imparts high flexibility for the movement of the antigen-binding domain. The first-generation CARs lacked the structure of CMs and led to poor replication, survival, and cytotoxicity of T cells. In contrast, the second- and third-generation CARs (Figure 1), with the addition of CMs, led to high proliferative capacity, long-term persistence, and potent cytotoxicity of T cells [35]. In the second-generation CARs, a CM (CM1), such as CD28, was engineered into the signaling region [36], whereas in the third generation, additional CMs (CM2), such as CD27, CD137/4-1BB, and CD134/OX40, were included [37]. In some tumors with a tremendous phenotypic heterogeneity, CAR T cells could target the tumor antigen and cause antigen-positive cell death, while antigen-negative cancer cells may induce tumor relapse. Recently, CAR T cells with a transgenic “payload,” also called the “fourth-generation” CAR T cells, were designed [38]. The fourth-generation CAR T cells work by releasing inducible cytokines such as IL-12 which will augment T cell activation and further activate innate immune system to kill antigen-negative cancer cells. Recently, CAR T cell therapy has received much attention as an immunotherapy for tumors, and a good efficacy has been reported in some clinical trials of leukemia and lymphoma. CAR T cell therapy is also being investigated for solid tumors, such as HCC. Glypican3 (GPC3) is a TAA that is specifically overexpressed in 70%–81% of HCC tumors and has been correlated with poor prognosis [39]. Moreover, the ability of GPC3-targeted CAR T cells to eliminate GPC3-positive HCC cells was confirmed both in vivo and in vitro, and the survival of mice with HCC xenografts was evidently prolonged with CAR T cell therapy in vivo [40]. In another experiment, T cells with two complementary CARs against GPC3 and asialoglycoprotein receptor 1 (ASGR1) decreased the risk of on-target, off-tumor toxicities and demonstrated potent antitumor immune responses targeting GPC3+ ASGR1+ HCCs both in vivo and in vitro [41]. However, to date, the related studies conducted have been predominantly basic, and more clinical trials are required to prove the efficacy of CAR T cells against HCC. Complications of CAR T cell therapy include on-target, off-tumor toxicities [42], tumor lysis syndrome (TLS) [43], and cytokine release syndrome (CRS) [44]. Traditional solutions include nonspecific immunosuppression, complete elimination of T cells, and introduction of inducible suicide genes into CAR T cells. However, the best method to prevent these hazards could be the application of ideal tumor-specific antigens (TSAs) expressed only in tumor cells and not in normal cells. As for the difficulty of seeking for more appropriate TSAs, some techniques, such as the utilization of inhibitory CAR (iCAR) [45] and combinatorial antigen recognition by CAR and chimeric costimulatory receptor (CCR) [46], were employed to prevent on-target, off-tumor toxicities. In addition, CARs could be used to modify other lymphocytes, such as NK cells [47] and *γδ*T cells [48], which may highlight the use of HCC immunotherapy in the future.

## 4. HCC Vaccines

Cancer vaccination is performed using antigenic substances to activate tumor-specific immune responses that can reduce tumor load and prevent tumor relapse. HCC vaccines include cancer cells, antigen peptides, DCs, and DNA-based vaccines, and some of these effectively inhibit tumor recurrence and metastasis.

### 4.1. HCC Cell Vaccines

Autologous or allogenic HCC cells or lysates that are physically or chemically disposed to eliminate pathogenicity could be used as immunogens for tumor-specific immune responses. In a phase I trial, bi-shRNA/granulocyte macrophage colony-stimulating factor- (GM-CSF-) augmented autologous tumor cells were tested in eight patients with advanced HCC. Three of these patients presented evident immune responses to the reinfused tumor cells, and long-term follow-up demonstrated a survival of 319, 729, 784, 931+, and 1043+ days after treatment [49]. However, the efficacy of HCC cell vaccines remains uncertain due to their weak immunogenicity.

### 4.2. Antigen Peptide Vaccines

Peptide-based TAAs, such as alpha-fetoprotein (AFP), GPC3, SSX-2, NY-ESO-1, human telomerase reverse transcriptase (hTERT), HCA587, and melanoma antigen gene-A (MAGE-A), are excellent vaccine targets for the treatment of HCC [50]. AFP, which normally originates from embryonic liver cells, can be overexpressed on HCC cell surfaces. However, immune responses to AFP are limited due to acquired immune tolerance during the development of the immune system. To break up this immune tolerance, a research group investigated the use of a recombinant rat AFP to induce cross-reactions between xenografts and endogenous molecules in animals and observed modest cellular and humoral immune responses [51]. In a phase II trial of GPC3-derived peptide vaccine for HCC, 25 patients received 10 vaccinations over one year after surgery. Recurrence in patients who underwent both surgery and vaccination was specifically lower than that in 21 patients who underwent surgery only (24% versus 48% and 52.4% versus 61.9% at 1 and 2 years, *p* = 0.047 and 0.387, resp.), indicating the efficacy of the GPC3-derived vaccine [52].

### 4.3. DC Vaccines

DCs, the most powerful APCs, are responsible for absorption, processing, and presentation of tumor antigens. They maintain high expression levels of MHCs and CMs, such as B7-1 and B7-2. They also elicit antitumor effects by the way of inducing primary T cells, releasing IFN-*γ* that suppresses tumoral angiogenesis and producing immune memory [53]. During vaccine preparation, DCs are initially activated by cytokines, such as rhGM-CSF and rhIL-4, then mature in the presence of tumor necrosis factor- (TNF-) *α* and are finally sensitized by autologous tumor cells or antigens [50]. Some gene-transfected DCs persistently express endogenous tumor antigens or cytokines that enhance their own functions. In a recent study, mice with HCC were treated with a combination of tumor cell lysate- (TCL-) loaded DCs and nifuroxazide, which is an inhibitor of signal transducer and activator of transcription 3 (STAT3). This combination increased the survival rate, limited tumor growth, and elevated antitumor immune response [54]. A phase I/IIa study using tumor antigen-pulsed DCs for HCC patients after primary treatment demonstrated that DC vaccination is an effective adjuvant treatment for such patients [55]. In addition, the safety and tolerance of DC vaccines have been confirmed in patients with HCC [56].

## 5. Immune Checkpoint Inhibitors

As mentioned above, the negative regulatory target-immune checkpoints are often overexpressed in tumors to escape the host immune surveillance. Immune checkpoint inhibitors can reactivate tumor-specific T cells and develop an antitumor effect by suppressing checkpoint-mediated signaling [57]. Common immune checkpoint proteins include cytotoxic T lymphocyte-associated antigen-4 (CTLA-4), programmed cell death protein-1 (PD-1), programmed cell death ligand 1 (PD-L1), VISTA, TIM-3, LAG-3, and OX40 [58, 59]. CTLA-4 and PD-1 inhibitors have been well characterized and have been approved by FDA for treating melanomas, with some progress in their application in treating HCCs.

### 5.1. CTLA-4 Inhibitors

CTLA-4 is predominantly expressed in activated T cells and NK cells [60]. It binds ligands B7-1 and B7-2 with much higher affinity than CD28 [61]. Moreover, CTLA-4 inhibitors prevent the binding of CTLA-4 to B7-1 and B7-2, thereby promoting the activation of T cells. In 2011, FDA approved a fully human anti-CTLA-4 mAb-Ipilimumab for the treatment of metastatic melanoma. In a phase II study of an anti-CTLA-4 mAb-Tremelimumab in patients with advanced HCC and hepatitis C, partial response rate (17.6%), disease control rate (76.4%), and time to progression (6.48 months) improved. Moreover, viral loads of HCC were significantly decreased, and no patients experienced immune-related adverse events (irAEs) or evident hepatotoxicity. These studies demonstrated that Tremelimumab treatment is a safe antitumor and antiviral method for hepatitis C-induced HCC [62]. In a noncomparative clinical trial involving patients with advanced HCC, a combination therapy with Tremelimumab and RFA increased the number of intratumoral CD8^+^T cells and reduced HCV viral loads [63].

### 5.2. PD-1 Inhibitors

PD-1 is expressed in T cells, B cells, NK cells, mononuclear cells, and DCs [64]. PD-1 inhibitors block the receptor binding of PD-L1 and PD-L2, resulting in the activation of immune cells [65]. Some PD-1 inhibitors, such as Nivolumab, Pembrolizumab, and Pidilizumab, have been investigated for cancer treatment. A phase I/II study demonstrated the safety and antitumor effect of Nivolumab in patients with advanced HCC. In this study, of the 41 qualified patients who were intravenously administered 0.1–10 mg/kg Nivolumab, 29 (71%, 17% grade 3/4) endured drug-related AEs, two (5%) showed complete responses (CRs), and seven (18%) showed partial responses (PRs). Moreover, response durations for CR, PR, and stable disease (SD) were 14–17+ months, <1–8+ months, and 1.5–17+ months, respectively, and the OS rate at 6 months was 72%. These data indicated that Nivolumab activates sustained tumor-specific immune responses with manageable AEs [66]. A recent open-label, noncomparative, phase I/II dose escalation and expansion trial of Nivolumab involving 262 patients with advanced HCC confirmed the safety and potential of this PD-1 inhibitor in treating HCCs [67].

### 5.3. PD-L1 Inhibitors

Cancer cells can evade immune surveillance by overexpressing PD-L1 and activating PD-L1/PD-1 signaling [68]. High PD-L1 expression has been observed in HCC tissues [69]. However, no clinical trials involving the use of PD-L1 inhibitors for treating HCC have been conducted. A recent experiment showed that contemporary inhibition of PD-L1 and DNA methyltransferase 1 (DNMT1) significantly suppressed the growth of sorafenib-resistant HCC cells in vitro, further suggesting a novel effective treatment option for sorafenib-resistant HCC [70].

## 6. Oncolytic Virotherapy

Oncolytic viruses are wild-type or engineered viruses that selectively replicate in tumor cells and cause lysis without harming normal tissues [71, 72]. The mechanism underlying the antitumor activity of oncolytic viruses involves direct killing of cancer cells by expanding in them and causing cell lysis. Most viruses can expand in cancer cells to a rather great extent due to the impairment of the tumor's defense mechanisms against viral infection [73]. In addition, tumor antigens and viruses in cell lysates activate immune responses against adjacent cancer cells [74–77]. The targeting mechanisms of oncolytic viruses are as follows. First, wild-type viruses that specifically infect tumors like reoviruses, varicella viruses, and Sindbis viruses [78] could be chosen. Second, viral genes that are crucial for replication in normal cells but have no functions in cancer cells are deleted by engineering [76]. Third, viral transcription is limited in cancer cells by applying tumor-specific promoters, such as the promoter of human telomerase reverse transcriptase, before crucial viral genes [79]. Finally, after modification by TAA-specific receptors, viruses effectively target tumor cells. For example, an oncolytic vaccinia virus engineered with antiangiogenic genes can specifically inhibit tumor angiogenesis [80]. The efficacy of an evolutionary cancer-favoring engineered vaccinia virus (CVV) was investigated in an animal model of metastatic HCC. In this study, animals were randomized into sorafenib, CVV, and sorafenib with CVV groups. Metastatic regions were fewer in the CVV-treated groups than in the sorafenib-treated group. The result suggested that CVV can be a promising virus targeting metastatic HCC [81]. JX-594, an engineered vaccinia virus with a mutation in the TK gene, which controls cancer cell-specific replication, and an insertion in the human GM-CSF gene, which increases antitumor immune responses [82], is stable and safe in humans and extremely toxic to cancer cells. A phase II randomized open-label study of JX-594 in patients with advanced HCC confirmed the safety and efficacy of the oncolytic virotherapy. This treatment was well tolerated at both high and low doses, with an intrahepatic response rate of 62% and one CR. In addition, the OS rate was higher in the high-dose group than in the low-dose group (median, 14.1 months versus 6.7 months; hazard ratio, 0.39; *p* = 0.020) [83]. To date, various oncolytic viruses, such as GLV-1h68 [84] and G47delta [85], have been studied for the treatment of HCC. Researchers should attach more importance to the dangers of viral infection and the insertional mutations that may activate oncogenes or damage tumor suppressor genes.

## 7. Brief Summary

The four major immunotherapeutic approaches for HCC have their own preponderances and defects.

CAR T cell therapy has been a star of immunotherapeutic researches in recent years. With its accurate targeting toward HCC and MHC independence, CAR T cells could directively kill HCC cells, like precision-guided missiles. The efficacy of CAR T cells has also been elevated after several generations. However, this favored method is not almighty. The lack of HCC-associated TSAs makes it difficult to construct more efficacious CARs. Meanwhile, more strategies should be designed to overcome the on-target, off-tumor effect. Other methods of adoptive cell therapy, like CIK cells, TILs, and NK cells, are being out of sight due to the nonspecificity and difficulty of extraction.

Immune checkpoint inhibitor is another hot topic. It breaks up tumor immune tolerance and causes reactivation of innate immune system, which may redirect and eliminate HCC cells as a result. It is a relatively simple process preparing for immune checkpoint inhibitors. Meanwhile, many clinical researches indicate the safety of this method. So, we may focus on how to improve its efficacy and test more practical combinatorial therapeutic methods in the future.

Tumor vaccines, because of tumor immune tolerance and lack of TSAs, did not show great value in HCC treatment, while DC vaccines may be a promising method in this realm, due to their potent capacity of antigen presenting. Researches of oncolytic viruses are quite few. Safety of viruses is the most important, while efficacy is the second. So the very much difficulty is to balance safety and toxicity of oncolytic viruses.

## 8. Future Expectations

As a new therapeutic approach for malignancies beyond traditional operations, chemotherapy and radiotherapy, immunotherapy has shown its efficacy in delaying the progression of advanced tumors and protecting postoperative patients against cancer relapse and metastasis. Although no drugs have been officially approved, numerous studies on immunotherapy for HCC are being conducted and some have already obtained important results. Future studies are required to identify more specific immune targets, such as TAAs/TSAs, novel immune checkpoints, and oncolytic viruses. These will enhance the intensity of tumor-specific immune responses and avoid unnecessary on-target, off-tumor toxicities. Meanwhile, the Aes should be valued, especially in clinical trials. The safety of a new treatment is as important as its efficacy. Furthermore, individualized treatment plans for patients with HCC will enhance the efficacy of immunotherapy and likely become a future trend. Taken together, the promising therapeutic approach certainly will bring the treatment for HCC to a brand new period.

## Figures and Tables

**Table 1:** Major immunotherapeutic approaches for HCC.

Approaches	Subsets	Targets and applications
ACT	CIK cells	CIK with valproate, DC-CIK with TACE
	TILs	
	NK cells	NK with K562-mb15-41BBL, sorafenib, and NKG2D
	CAR T cells (generations 1–4)	Targeting GPC3, targeting GPC3 and ASGR1

HCC vaccines	Cell vaccines	HCC cells with GM-CSF
	Antigen peptide vaccines	AFP, GPC3, SSX-2, NY-ESO-1, hTERT, HCA587, and MAGE-A
	DC vaccines	TCL-loaded DCs with nifuroxazide

Immune checkpoint inhibitors	CTLA-4 inhibitors	Tremelimumab, Tremelimumab with RFA
	PD-1 inhibitors	Nivolumab, Pembrolizumab, and Pidilizumab
	PD-L1 inhibitors	PD-L1 inhibitor with DNMT1 inhibitor

Oncolytic viruses		CVV, JX-594, GLV-1h68, and G47delta
